# Surface Modification of Nanocrystalline LiMn_2_O_4_ Using Graphene Oxide Flakes

**DOI:** 10.3390/ma14154134

**Published:** 2021-07-24

**Authors:** Monika Michalska, Dominika A. Buchberger, Jacek B. Jasiński, Arjun K. Thapa, Amrita Jain

**Affiliations:** 1 Department of Chemistry, Faculty of Materials Science and Technology, VŠB-Technical University of Ostrava, 17. Listopadu 2172/15, 708 00 Ostrava-Poruba, Czech Republic; 2 Łukasiewicz Research Network—Institute of Microelectronics and Photonics, Al. Lotników 32/46, 02-668 Warsaw, Poland; 3 Faculty of Chemistry, University of Warsaw, Pasteura 1, 02-093 Warsaw, Poland; daziolkowska.edu@gmail.com; 4 Conn Center for Renewable Energy Research, University of Louisville, Louisville, KY 40292, USA; jbjasinski@gmail.com (J.B.J.); akthap01@louisville.edu (A.K.T.); 5 Institute of Fundamental Technological Research, Polish Academy of Sciences, Pawińskiego 5B, 02-106 Warsaw, Poland; ajain@ippt.pan.pl

**Keywords:** lithium manganese oxide, LiMn_2_O_4_, graphene oxide, cathode material, lithium ion battery

## Abstract

In this work, a facile, wet chemical synthesis was utilized to achieve a series of lithium manganese oxide (LiMn_2_O_4_, (LMO) with 1–5%wt. graphene oxide (GO) composites. The average crystallite sizes estimated by the Rietveld method of LMO/GO nanocomposites were in the range of 18–27 nm. The electrochemical performance was studied using CR2013 coin-type cell batteries prepared from pristine LMO material and LMO modified with 5%wt. GO. Synthesized materials were tested as positive electrodes for Li-ion batteries in the voltage range between 3.0 and 4.3 V at room temperature. The specific discharge capacity after 100 cycles for LMO and LMO/5%wt. GO were 84 and 83 mAh g^−1^, respectively. The LMO material modified with 5%wt. of graphene oxide flakes retained more than 91% of its initial specific capacity, as compared with the 86% of pristine LMO material.

## 1. Introduction

The discovery of new innovative materials as well as adding new functionalities to well-known materials using various “bottom up” and “top down” techniques is highly important from the practical point of view for various applications, including the energy sector [1,2,3,4,5,6,7]. The development of energy technologies, especially renewable energy and energy storage systems will play a key role in urgently needed de-carbonization and transition to a low-emission economy. The importance of these technologies has been recognized in recent years. For example, the Nobel Prize in Chemistry 2019 (John B. Goodenough, M. Stanley Whittingham, and Akira Yoshino) was awarded for the development of lithium-ion batteries (LiBs) and for the first time for an application commonly used in portable electronic devices, such as mobile phones, laptops, different gadgets, and electric cars. LiBs are also used to store energy from renewable sources, such as solar and wind power [8,9,10,11,12,13,14,15,16,17].

Cubic lithium manganese oxide (LiMn_2_O_4_, LMO) is recognized as an attractive candidate for a positive electrode material in lithium ion batteries and supercapacitors [12,16,17,18,19]. Its uniqueness lies in its simplicity. In particular, it is non-toxic, low-cost, easy to prepare, possesses high discharge potential (4.1 V vs. Li metal), and is environmentally friendly compared to other commercially-viable cathode materials, such as layered lithium cobalt (LiCoO_2_) or lithium nickel (LiNiO_2_) oxides [18,19,20,21,22]. However, this material has disadvantages, particularly capacity fading during charge-discharge cycles at higher voltages than 4.1 V (vs. Li/Li^+^), especially at elevated temperature regions of 50–60 °C, which limits the use of LMO in commercial LiBs [23,24,25]. This problem originates from: (1) a cooperative Jahn-Teller transition effect from cubic to hexagonal structure, which generates strains and fracturing, and (2) Mn loss caused by disproportional reaction 2Mn^3+^ -> Mn^4+^ + Mn^2+^ followed by Mn^2+^ dissolution into the commonly used standard liquid electrolyte [12,24,25]. These are the challenges that need to be addressed for a large-scale commercial use of this material. One of the promising strategies is surface modification of LMO grains with formation of a coating or thin film layers such as carbon, metallic, or ceramic oxides [24,25,26,27,28,29,30,31,32,33,34,35,36,37,38,39,40,41]. This kind of modification can prevent direct contact between the electrolyte solution and the electrode material, helping to improve structural stability and suppress phase transitions [24,25,26,27,28,29,30,31,32,33,34,35,36,37,38,39,40,41]. With its superior electrical characteristics, graphene has recently become the most sought-after addition for electrode materials. It is usually used in the form as graphene oxide (GO) or reduced graphene oxide (RGO), which enables the creation of composites with a variety of materials. With the addition of graphene material to LiMn_2_O_4_ synthesis, fine, homogeneous, and non-agglomerated powders with small particles and outstanding electrochemical characteristics are typically obtained. A one-step hydrothermal approach without thermal treatment to synthesize LMO/graphene nanosheets (GNS) has been proposed by B. Lin et al. [42]. Their as-synthesized LMO/GNS nanocomposite showed good cathode performance with high specific capacity, good cycling stability, and rate capability as compared with the pristine LMO [42]. K.-Y. Jo and co-authors [43] used the solvothermal route to modify the LMO surface with reduced graphene oxide (RGO) nanosheets. Their prepared composite material exhibited high ionic diffusivity and electrochemical performance [43]. Y. Chen et al. [44] obtained an LMO/RGO nanocomposite using a low-temperature solvothermal process. They produced composites with 137.5 mAh g^−1^ of initial discharge capacity at a 0.5 C rate, while after 200 charging and discharging cycles, 75.6% of initial capacity remained [44]. Hybrid materials composed of LMO/RGO was synthesized by a microwave-assisted hydrothermal method by S.-M. Bak et al. [45]. The obtained material revealed a high specific capacity of 137, 117, and 101 mAh g^−1^ at 1 C, 50 C, and 100 C rates, respectively [45]. The precipitation synthesis was developed to modify LiMn_2_O_4_ with graphene by A. Li and co-authors [46]. The graphene-modified LMO material achieved 127 mAh g^−1^ of initial discharge capacity, while the after 100 charging and discharging cycles capacity retention rate was 96.2% [46]. All the aforementioned works [42,43,44,45,46] have claimed that coating LiMn_2_O_4_ with graphene led to the enhanced electrochemical performances due to the establishment of fast Li^+^ channels and improved structural stability of the LMO material.

The main challenge in the battery technology is to achieve the highest capacity but it has yet to be reached as expected. In the present study, we have tried to modify the materials to address this issue. Herein, we present a facile, wet chemical approach based on a simple, wet chemical, low-temperature process of surface modification of LMO grains using graphene oxide flakes. A lithium-manganese oxide powder was first synthesized using a modified sol-gel method [20,21,22,23,24,25]. Then, such pre-synthesized LMO powder was coated with GO flakes using a low-temperature process to obtain LMO/GO composites. For the first time in this work, we demonstrate the wet chemical and low temperature technique of the surface modification of the cathode material LiMn_2_O_4_ with graphene oxide flakes. Contrary to other studies, our technique is relatively fast, non-toxic, and cost-effective. The synthesis was conducted without the use of advanced and expensive equipment, such as autoclave used for the hydrothermal or solvothermal synthesis. After the surface modification of LMO material with GO flakes, no destruction of the LMO structure was observed. All synthesized materials were characterized extensively by a number of methods, including X-ray powder diffraction (XRD), Raman spectroscopy, and scanning and transmission electron microscopy (SEM, TEM). Electrochemical tests were performed for pristine LMO and LMO modified with 5%wt. of graphene oxide.

## 2. Materials and Methods

### 2.1. Synthesis of LMO Modified with 1–5%wt. of Graphene Oxide Flakes

**Figure 1:**
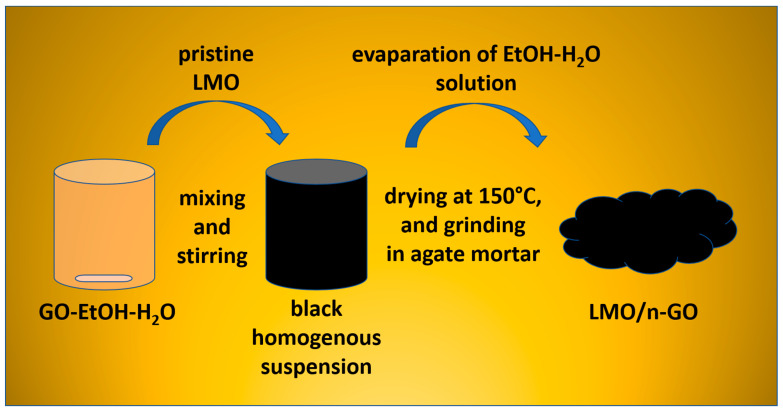
The flowchart of facile, wet chemical synthesis of LMO/1–5%wt. GO.

A modified sol-gel method was applied to create nanocrystalline LiMn_2_O_4_ powder using citric and acetic acids as chelating agents. The synthesis description of LMO material was demonstrated in our earlier work [20,21,22,23,24,25]. The modified Hummers technique was utilized to obtain a graphene oxide aqueous suspension [47,48,49,50]. A facile, wet chemical synthesis was proposed to modify LMO surface with graphene oxide flakes. At the first stage of synthesis, the as-prepared LMO nanocrystalline powder was dispersed in ethanol (96% pure p.a., CHEMPUR) solution to achieve a black suspension. Next, the LMO-EtOH-H_2_O suspension GO was added. This part of the synthesis was performed for 4 h under constant magnetic stirring at room temperature to obtain a homogenously dispersed suspension. Then, the EtOH-H_2_O solution was slowly evaporated at 60 °C for 12 h. Afterward, the lithium manganese oxide with graphene oxide (1–5%wt. GO) samples were air-dried overnight at 150 °C. LMO/1–5% GO fine powders were reached after grinding all materials in agate mortar. The flowchart of facile, wet chemical synthesis is presented in Figure 1.

### 2.2. Characterization of LMO/1–5%wt. GO Materials

The structural properties of LMO/1–5%wt. GO materials were investigated using powder X-ray diffraction (XRD) and Raman spectroscopy at room temperature. A SIEMENS D500 diffractometer (München, Germany) equipped with a Cu Kα (λ_XRD_ = 1.542 Å) radiation source was used to perform the structural analysis in order to identify the crystal structure, determine the crystallite sizes, and measure unit cell parameters of as-synthesized LMO/1–5%wt. GO samples. The XRD patterns in the range of 15° ≤ 2θ ≤ 60° were registered with a step size of 0.002° and acquisition time of 3 s per step. The average size of crystallites *d* was determined from the linewidths of XRD peaks using the Scherrer Formula (1) in its simplest form, assuming that XRD peak broadening was solely dependent on crystallite size [21].

*d* = *Kλ*(*βcosθ*)^−1^ (1)

where *K* is a shape factor between 0.9 and 1.1, λ_CuKα_ is the incident X-ray wavelength (here CuK_α_ = 1.542 Å), *β* is the full width at half-maximum (FWHM) of the selected peak, and *θ* is the Bragg’s angle of the peak.

The Raman spectra were collected using Renishaw in Via Raman Microscope (Charfield, UK) equipped with a 532 nm emission line of Nd:YAG laser. The surface morphology and particle size of synthesized LMO/1–5%wt. GO powders was examined by using a Carl Zeiss CrossBeam Auriga (Oberkochen, Germany) scanning electron microscope (SEM). The powders were also analyzed using a FEI Tecnai F20 (Hillsboro, OR, USA) transmission electron microscope (TEM), operating at 200 kV accelerating voltage. TEM specimens were prepared by dispersing sample powders on commercial holey carbon-coated TEM copper grids.

### 2.3. Electrochemical Studies

The electrochemical characterization was performed using CR2013 coin-type cells assembled in a dry argon-filled glove box. The cathodes were fabricated by mixing 10 mg of active electrode with 3 mg of teflonized acetylene black (TAB-2) as a conducting binder. The mixture was pressed onto stainless steel mesh. The average thickness of each cathode was 18–20 µm and Li foil was used as a counter electrode separated by a porous propylene film (ADVANTEC GB-100R). The cathodes were dried at 150 °C for 5 h under vacuum. The electrolyte used was 1 M LiPF_6_-EC:DMC (1:2). Every cell was cycled using the constant current mode in a potential range between 3.0 V and 4.3 V at room temperature using VMP3 Bio-Logic Science Instruments (Seyssinet-Pariset, France).

## 3. Results

### 3.1. XRD and Raman Spectra

**Figure 2:**
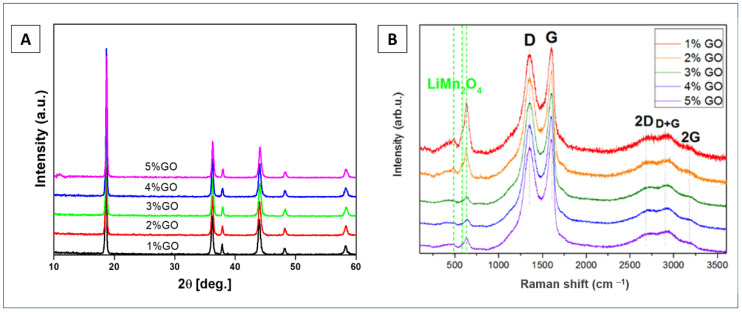
The XRD results (**A**) and Raman spectra (**B**) of LMO/1–5%wt. GO.

Figure 2A presents the XRD pattern of series of LMO nanocrystalline powders surface modified with 1–5%wt. of graphene oxide. The XRD pattern shown in Figure 2A reveals six characteristic peaks located at: 18.7°, 36.3°, 37.9°, 44.2°, 48.4°, and 58.4°, which correspond to the (111), (311), (222), (400), (422), and (511) crystal planes, respectively [20,21,22]. Their positions are characteristic of the cubic spinel crystal structure with Fd3m space group. The obtained unit cell parameters between 8.214 and 8.226 Å for all LMO/1–5%wt. GO powders closely match the standard value a_0_ = 8.24762 Å (V_0_ = 561.03 Å) for lithium manganese oxide (ICDD PDF-35-0782). The lattice parameter values measured for LMO/GO composites are presented in Table 1. They all are within the 8.206–8.251 Å, i.e., the range of LMO spinel data reported in the literature and ICDD database [22]. The measured small differences in the lattice constants could be due to variations in the actual stoichiometry (partial mixing of the positions of Li and Mn cations and possible vacancies in the positions of cations and oxygen) [22]. The analysis of peak broadening using the Scherrer formula estimated the average crystallite size, which for all studied samples, was found to be between 18 to 27 nm. The (511) XRD peak located at a relatively large 2θ angle was chosen for the Scherrer’s analysis to assure relatively small errors from the geometry of the measurement system. A crystallite or a crystalline grain is a single crystal domain of a powder that gives rise to a coherent scattering of the X-ray beam. A particle, on the other hand, may consist of many crystallites. Based on the XRD measurements, the mean crystallite size is determined, while the mean particle size can be determined based on the SEM measurements (see below, Section 3.2). Following the Scherrer’s formula, the crystallite size affects the width and intensity of the diffraction peak. The smaller the size of the crystallites, the greater the broadening of the peak. The discrepancies in the estimated crystallite sizes obtained from the Scherrer’s formula for all investigated samples could be due to the fact that total XRD peak broadening is influenced not only by grain size but also by instrumental broadening. The instrumental broadening may dominate for samples with relatively large crystallites, which seem to be the case for our powders. Table 1 summarizes the results of XRD analysis (crystallite size, lattice parameter, and cell volume).

Expectedly, the crystal structure of all LiMn_2_O_4_ powders was not changed after modification with graphene oxide. Raman analysis for all LMO/1–5%wt. GO powders (Figure 2B) showed a typical spinel spectrum of LiMn_2_O_4_ material (in the range of 100–700 cm^−1^) [23,24,25] as well as relatively sharp D and G peaks of graphene oxide structure (in the range of 1000–3500 cm^−1^) [44]. The characteristic bands with assignments for LMO and GO are summarized in Table 2.

The LiMn_2_O_4_ spinel crystal structure has five optical Raman active modes (see Table 2) [23,24,25,51,52]. A symmetric Mn–O stretching vibration of MnO_6_ groups contributes the strongest visible band of LMO at 636 cm^−1^, which is associated with the A_1g_ mode. T_2g_^(3)^ and T_2g_^(2)^ are attributed to large oxygen movements and very small Li ion displacements, respectively [23,24,25,51,52]. The well-visible A_1g_ shoulder peak at 581 cm^−1^ and the following weaker band at 491 cm^−1^ are assigned to large oxygen movements and very small Li ion displacements, respectively [23,24,25,51,52]. The T_2g_^(1)^ phonon, which is assumed to be the vibrations of Li sublattice, is associated with the visible band at 385 cm^−1^ [23,24,25,51,52].

Seven bands were registered in the graphene oxide structure at 1150, 1350, 1490, 1595, 2696, 2945, and 3180 cm^−1^ (see Table 2) [44,53,54]. A, D, and G lines represented the most intensive peaks located at 1350 and 1595 cm^−1^. The most intense G line was located owing to a doubly degenerate E_2g_ phonon mode active for sp^2^ carbon networks [44,53,54]. D line was assigned to the zone-boundary (K point) phonon due to the breathing modes (A_1g_ symmetry) of “honey-combed” carbon rings, or in a graphitic structure originated in the disorder due to limited crystallite size and defects [44,53,54]. The phonon mode at M point in the Brillouin zone, or C=O vibrations of surface oxidized areas, was thought to be the source of the peak at 1490 cm^−1^ between the D and G bands [44,53,54]. A second order phonon mode was an overtone of the D band (2 × 1348 cm^−1^), and corresponded to the 2D peak at 2696 cm^−1^ [44,53,54]. A combination of D and G peaks located at 2945 cm^−1^ could be induced by disorder or assigned to the sp^2^ and sp^3^ C-H stretching vibrations [44,53,54]. The overtone of the G band, or the stretching vibrations of C-OH groups, is represented by the line at 3180 cm^−1^ [44,53,54]. In addition, Raman spectroscopy analysis also showed that with increasing content of graphene oxide from 1 to 5 wt.%, the intensity of the strongest characteristic peak of the cubic spinel LMO structure band, located at 636 cm^−1^, decreased. This suggests that the graphene oxide covered the surface LMO particles. This was further confirmed by scanning electron (SEM) as well as transmission electron microscopy (TEM) analyses.

### 3.2. SEM and TEM Morphology Data

**Figure 3:**
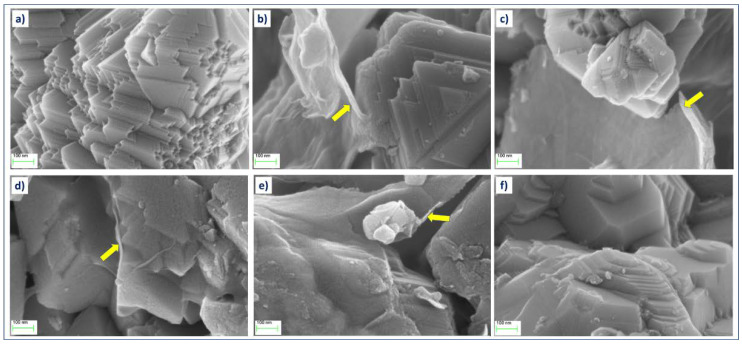
SEM images of (**a**) LMO powder with a various content of GO: (**b**) 1%wt., (**c**) 2%wt., (**d**) 3%wt., (**e**) 4%wt., (**f**) and 5%wt.

**Figure 4:**
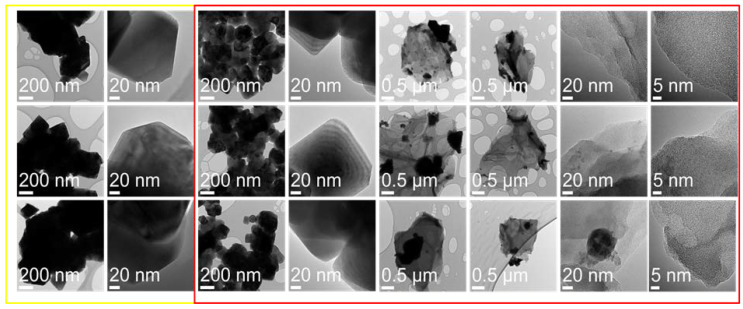
TEM images of LMO (yellow) and LMO with 5%wt. GO (red).

Figure 3 presents SEM images obtained at the same magnification (100,000×) from LiMn_2_O_4_ powders with various contents of graphene oxide. On the powder LMO grains, crystal growth planes are clearly visible with a size distribution between 100 and 600 nm, which agglomerate into larger particles. While the morphology of LMO grains in all these samples remains practically the same, additional features in a form of folded thin coating layers (some marked with yellow arrows) can be noticed in the samples with the addition of GO flakes. Such GO flakes attached to LMO particles are better visible in TEM images obtained from these samples (Figure 4). The overall morphology of the flakes can be best seen in low-magnification TEM images. However, high-resolution TEM images of these flakes clearly show the amorphous-like structure of these flakes (see images in the last column in Figure 4).

**Figure 5:**
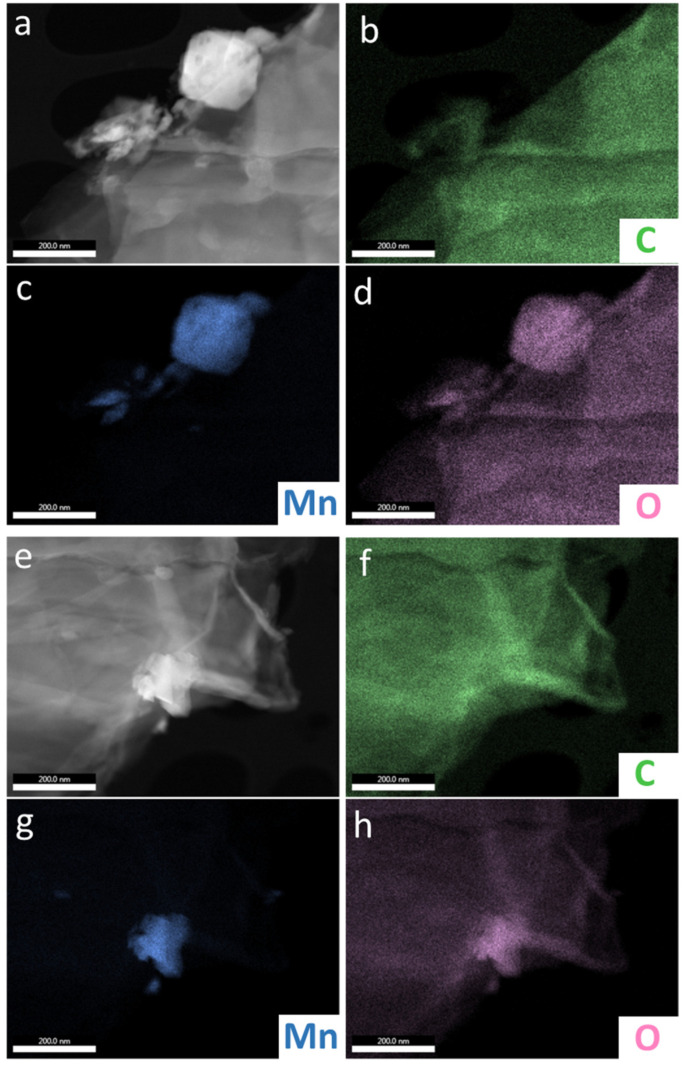
EDS analysis of LMO with 5%wt. GO composite: STEM images (**a**,**e**) and corresponding carbon (**b**,**f**), manganese (**c**,**g**), and oxygen (**d**,**h**) maps measured at two different regions of the sample.

The EDS analysis for manganese (Mn), oxygen (O), and carbon (C) elements was performed for sample LMO with 5%wt. GO to confirm the presence of a carbon structure on LMO surface grains. The results are depicted in Figure 5. It may be that graphene oxide flakes are covering the LMO grains.

### 3.3. Electrochemical Performances of LMO and LMO/5%GO Materials

**Figure 6:**
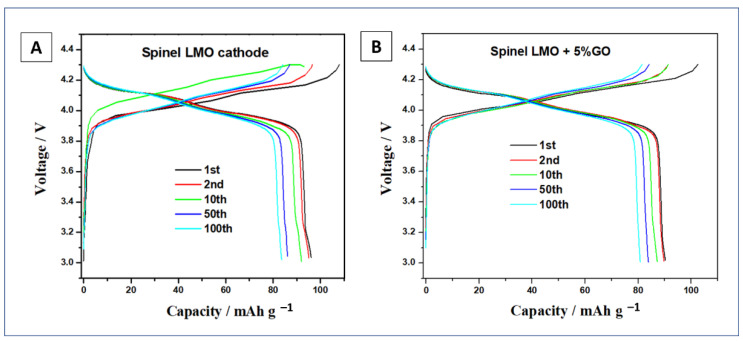
Charge-discharge curves of LMO (**A**) and LMO + 5%GO; (**B**) spinel electrode for Li-ion battery at the voltage range of 4.3–3.0 V with a current of 20 mA g^−1^.

Two materials were chosen for electrochemical performance testing, namely pristine LMO and LMO modified with 5%wt. graphene oxide. The galvanostatic charge-discharge profiles of the positive electrodes were measured using constant current mode in a potential range between 3.0 and 4.3 V at room temperature (see Figure 6). The samples were examined using a CR2013 coin-type cell. The galvanostatic charge curves show two characteristic plateaus, at potentials of about 4.0 and 4.2 V, corresponding to the subsequent stages of LMO oxidation. During the discharge of the studied cells, a two-stage reduction process, with plateaus appearing at about 4.1 and 3.9 V, characteristic for LiMn_2_O_4_ material, was observed. At 20 mA g^−1^ current density, in the first and second discharge process, the pristine LMO and with 5%wt. GO electrode showed a specific capacity of 98, 91, and 95, 90 mAh g^−1^, respectively. The specific capacities of the cells discharged at 20 mA g^−1^ current rate after the 10th and 50th cycles were: 93, 87, and 87, 84 mAh g^−1^, for LMO and LMO/5%wt. GO, respectively. After 100 cycles of charge/discharge processes, both electrodes revealed similar values of specific capacity of 84 and 83 mAhg^−1^ for pristine LMO and modified with 5%wt. of graphene oxide, respectively.

**Figure 7:**
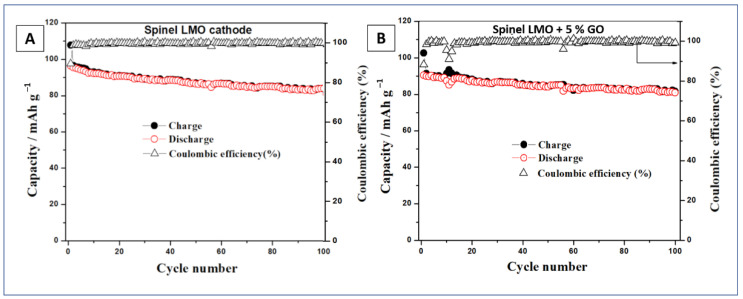
Cyclic performance and coulombic efficiency of LMO (**A**) and LMO + 5%GO; (**B**) spinel electrode for Li-ion battery at the voltage range of 4.3–3.0 V with a current of 20 mA g^−1^.

Figure 7 depicts the results of charge-discharge capacities vs. cycle number tests of LMO and LMO/5%wt. GO electrodes. After 100 cycles of charging and discharging tests (at 20 mA g^−1^ current rate) LMO/5%wt. GO material retained 91.2% of first discharge capacity, while the pristine LiMn_2_O_4_ showed only 85.7%.

The coulombic efficiency of both analyzed electrodes was at the same level between 99% and 100%. The achieved cyclability tests at 20 mA g^−1^ current density are summarized in Table 3.

The surface modification with graphene oxide led to improved cyclability of LMO powder. Despite the observed slightly lower values of specific capacity in all cycles for the LMO/5%wt. GO material as compared with the pristine LMO, the decrease in capacity in each cycle was smaller. As a result, a positive effect of surface modification of LMO grains with graphene oxide flakes was observed. To modify the surface of LiMn_2_O_4_ grains, graphene oxide (GO) material, which contained various functional groups such as C-O hydroxyl and epoxy, O-C=O carboxyl, carbonyl functional groups, as well as C=C/C-C in aromatic rings, was utilized [48,49]. Depending on the form of the graphene material used to modify the LMO surface, the electrochemical properties were positively influenced by the material, which was either in the form of pure graphene or in the form of reduced graphene oxide. Chen et al. [44] used a low-temperature solvothermal method to produce an LMO/RGO nanocomposite. At a 0.5 C rate, they developed a composite with 137.5 mAh g^−1^ of initial discharge capacity, with 75.6% of initial capacity remaining after 200 charging and discharging cycles [44]. S.-M. Bak et al. developed a hybrid material composed of LMO/RGO using a microwave-assisted hydrothermal technique [45]. At 1, 50, and 100 C rates, the material obtained throughout this approach revealed a high specific capacity of 137, 117, and 101 mAh g^−1^, respectively [45]. A. Li and co-authors proposed modifying LiMn_2_O_4_ with graphene using a precipitation synthesis [46]. The initial discharge capacity of the graphene-modified LMO material was 127 mAh g^−1^, with a capacity retention rate of 96.2% after 100 charging and discharging cycles [46]. The obtained results in our study seem to be promising, taking into account the relatively fast, non-toxic, and cost-effective chemical approach used for the modification of the LMO surface with graphene oxide flakes. The LMO material with the highest content of GO (5%wt.) had the smallest average crystallite sizes. Comparing our materials with other works [42,43,44,45,46], the discharge capacity values were not high, which on the other side had a positive effect on the values of capacity retained after 100 charging and discharging cycles, and as a result, almost 100% of coulombic efficiency was revealed. This, in turn, indicated that by modifying the surface of the LMO material with graphene oxide flakes, the effect of shortening the transport path of electrons and Li ions (between the electrode and electrolyte) should be reached. We demonstrated here the preliminary EIS results, since it was expected to work with reduced graphene oxide (more conductive material), which possesses less functional groups, as shown in other studies [42,43,44,45,46], and in turn was possible to attain better electrochemical results, such as specific capacity and cyclability.

## 4. Conclusions

In this work, we reported a facile, wet chemical synthesis of surface modification of LMO grains with graphene oxide flakes. The XRD results showed that LiMn_2_O_4_ crystallizes in the cubic spinel structure with the Fd3m space group. The LMO structure remained intact after the surface modification by GO coatings. Raman analysis showed a typical spinel feature of LiMn_2_O_4_ and relatively sharp D and G peaks of graphene oxide flakes. The electrochemical characterization was performed using a CR2013 coin-type cell configuration. The specific discharge capacity after 100 cycles for LMO and LMO/5%wt. GO were 84 and 83 mAh g^−1^, respectively. The LMO material modified with 5%wt. of graphene oxide flakes retained more than 91% of its initial specific capacity, as compared with 86% measured for pristine LMO material. In the future, it is planned to modify such a series of samples to produce LMO with graphene oxide in the reduced form. It is expected that after the reduction of certain functional groups of graphene oxide, a better electrochemical performance can be achieved while maintaining a high level of coulombic efficiency.

## Figures and Tables

**Table 1:** Average crystallite size, lattice parameter, and cell volume for LMO/1–5%wt. GO.

Sample	Average Crystallite Size (XRD), *d* [nm]	Lattice Parameter, *a* [Ẩ]	Cell Volume, *V* [Ẩ^3^]
LMO/1%wt. GO	27	8.226	556.6
LMO/2%wt. GO	21	8.225	556.4
LMO/3%wt. GO	20	8.214	554.1
LMO/4%wt. GO	26	8.221	553.4
LMO/5%wt. GO	18	8.221	555.6

**Table 2:** The band assignment of spectra of LMO/1–5%wt. GO.

Raman	Assignment
385 cm^−1^	related to the T_2g_^(1)^ phonon, considered as the vibrations of Li sublattice
581 and 491 cm^−1^	two bands are assigned to T_2g_^(3)^ and T_2g_^(2)^ respectively and originating from large oxygen motions and very small Li ion displacements
636 cm^−1^	the strongest visible band of LMO, related to the A_1g_ mode which consisted of a symmetric Mn–O stretching vibration of MnO_6_ groups
1150 cm^−1^	weak peak can be assigned to the phonon at K point of the graphite Brillouin zone
1350 cm^−1^	D line, the zone-boundary (K point) phonon due to the breathing modes (A_1g_ symmetry) of “honeycombed” carbon rings, or in a graphitic structure originated in the disorder due to limited crystallite size and defect
1490 cm^−1^	located between D and G bands attributed to the phonon mode at M point in Brillouin zone, or to C=O vibrations of surface oxidized regions
1595 cm^−1^	G line, a doubly degenerate E_2g_ phonon mode active for sp^2^ carbon networks
2696 cm^−1^	2D peak, a second order phonon mode (an overtone of the D band)
2945 cm^−1^	combination of D and G peaks can be induced by disorder, or assigned to the sp^2^ and sp^3^ C-H stretching vibrations
3180 cm^−1^	the overtone of the G band, or the stretching vibrations of C-OH groups

**Table 3:** Comparison of cyclability of LMO and LMO/5%wt. GO samples.

Sample	LMO	LMO/5%wt. GO
1st discharge capacity, mAh g^−1^	98	91
2nd discharge capacity, mAh g^−1^	95	90
10th discharge capacity, mAh g^−1^	93	87
50th discharge capacity, mAh g^−1^	87	84
100th discharge capacity, mAh g^−1^	84	83
Capacity retained after 100 cycles, %	85.7	91.2

## Data Availability

All data included in this study are available upon request by contact with the corresponding author.

## Abbreviations

LMO	lithium manganese oxide
GO	graphene oxide
TAB-2	teflonized acetylene black
EC	ethylene carbonate
DMC	dimethyl carbonate
LiPF_6_	lithium hexafluorophosphate
EC	ethylene carbonate
EtOH	ethanol
Li	lithium

## References

[1] Jiang C., Hosono E., Zhou H. (2006). Nanomaterials for lithium ion batteries. Nano Today.

[2] Lee K.T., Cho J. (2011). Role of nanosize in lithium reactive nanomaterials for lithium-ion batteries. Nano Today.

[3] Wang Y., Li H., He P., Hosono E., Zhou H. (2010). Nano active materials for lithium-ion batteries. Nanoscale.

[4] Song H.-K., Lee K.T., Kim M.G., Nazar L.F., Cho J. (2010). Recent Progress in Nanostructured Cathode Materials for Lithium Secondary Batteries. Adv. Funct. Mater..

[5] Sun Y.-K., Chen Z., Noh H.-J., Lee D.-J., Jung H.-G., Ren Y., Wang S., Yoon C.S., Myung S.-T., Amine K. (2012). Nanostructured high-energy cathode materials for advanced lithium batteries. Nat. Mater..

[6] Fergus J.W. (2010). Recent developments in cathode materials for lithium ion batteries. J. Power Sources.

[7] Mauger A., Julien C. (2014). Surface modifications of electrode materials for lithium-ion batteries: Status and trends. Ionics.

[8] Ramström O. (2019). Scientific Background on the Nobel Prize in Chemistry 2019: Lithium-Ion Batteries.

[9] Manthiram A. (2020). A reflection on lithium-ion battery cathode chemistry. Nat. Commun..

[10] Ramanan A. (2019). Nobel Prize in Chemistry 2019. Resonance.

[11] Kamat P.V. (2019). Lithium-Ion Batteries and Beyond: Celebrating the 2019 Nobel Prize in Chemistry—A Virtual Issue. ACS Energy Lett..

[12] Thackeray M.M. (2021). Exploiting the Spinel Structure for Li-ion Battery Applications: A Tribute to John B. Goodenough. Adv. Energy Mater..

[13] Nitta N., Wu F., Lee J.T., Yushin G. (2015). Li-ion battery materials: Present and future. Mater. Today.

[14] Tarascon J.-M., Armand M. (2001). Issues and challenges facing rechargeable lithium batteries. Nature.

[15] Armand M., Tarascon J.-M. (2008). Building better batteries. Nature.

[16] Whittingham M.S. (2004). Lithium Batteries and Cathode Materials. Chem. Rev..

[17] Thackeray M.M., David W.I.F., Bruce P.G., Goodenough J.B. (1983). Lithium insertion into manganese spinels. Mater. Res. Bull..

[18] Cai Y., Huang Y., Wang X., Jia D., Pang W., Guo Z., Du Y., Tang X. (2015). Facile synthesis of LiMn_2_O_4_ octahedral nanoparticles as cathode materials for high capacity lithium ion batteries with long cycle life. J. Power Sources.

[19] Xi L.J., Wang H.-E., Lu Z.G., Yang S.L., Ma R.G., Deng J.Q., Ching C.Y. (2012). Facile synthesis of porous LiMn_2_O_4_ spheres as positive electrode for high-power lithium ion batteries. J. Power Sources.

[20] Michalska M., Lipińska L., Sikora A., Ziółkowska D., Korona K.P., Andrzejczuk M. (2015). Structural and morphological studies of manganese based cathode materials for lithium ion batteries. J. Alloys Compd..

[21] Michalska M., Lipińska L., Mirkowska M., Aksienionek M., Diduszko R., Wasiucionek M. (2011). Nanocrystalline lithium-manganese oxide spinels for Li-ion batteries–sol-gel synthesis and characterization of their structure and selected physical properties. Solid State Ion..

[22] Michalska M., Lipińska L., Diduszko R., Mazurkiewicz M., Małolepszy A., Stobinski L., Kurzydłowski K.J. (2011). Chemical syntheses of nanocrystalline lithium manganese oxide spinel. Phys. Status Solidi C.

[23] Hamankiewicz B., Michalska M., Krajewski M., Ziolkowska D., Lipińska L., Kamińska M., Czerwinski A. (2014). The effect of electrode thickness on electrochemical performance of LiMn_2_O_4_ cathode synthesized by modified sol gel method. Solid State Ion..

[24] Michalska M., Hamankiewicz B., Ziółkowska D., Krajewski M., Lipińska L., Andrzejczuk M., Czerwiński A. (2014). Influence of LiMn_2_O_4_ modification with CeO_2_ on electrode performance. Electrochim. Acta.

[25] Michalska M., Ziółkowska D.A., Jasiński J.B., Lee P.H., Ławniczak P., Andrzejewski B., Ostrowski A., Bednarski W., Wu S.H., Lin J.Y. (2018). Improved electrochemical performance of LiMn_2_O_4_ cathode material by Ce doping. Electrochim. Acta.

[26] Zhao H., Li Y., Shen D., Yin Q., Ran Q. (2020). Significantly enhanced electrochemical properties of LiMn_2_O_4_-based composite microspheres embedded with nano-carbon black particles. J. Mater. Res. Technol..

[27] Tang M., Yuan A., Xu J. (2015). Synthesis of highly crystalline LiMn_2_O_4_/multiwalled carbon nanotube composite material with high performance as lithium-ion battery cathode via an improved two-step approach. Electrochim. Acta.

[28] Tang M., Yuan A., Zhao H., Xu J. (2013). High-performance LiMn_2_O_4_ with enwrapped segmented carbon nanotubes as cathode material for energy storage. J. Power Sources.

[29] Liu X.-M., Huang Z.-D., Oh S., Ma P.-C., Chan P.C.H., Vedam G.K., Kang K., Kim J.-K. (2010). Sol–gel synthesis of multiwalled carbon nanotube-LiMn_2_O_4_ nanocomposites as cathode materials for Li-ion batteries. J. Power Sources.

[30] Wang J., Zhang Q., Li X., Wang Z., Zhang K., Guo H., Yan G., Huang B., He Z. (2013). A graphite functional layer covering the surface of LiMn_2_O_4_ electrode to improve its electrochemical performance. Electrochem. Commun..

[31] Kumar N., Rodriguez J.R., Pol V.G., Sen A. (2019). Facile synthesis of 2D graphene oxide sheet enveloping ultrafine 1D LiMn_2_O_4_ as interconnected framework to enhance cathodic property for Li-ion battery. Appl. Surf. Sci..

[32] Son J.T., Park K.S., Kim H.G., Chung H.T. (2004). Surface-modification of LiMn_2_O_4_ with a silver-metal coating. J. Power Sources.

[33] Yu L., Qiu X., Xi J., Zhu W., Chen L. (2006). Enhanced high-potential and elevated-temperature cycling stability of LiMn_2_O_4_ cathode by TiO_2_ modification for Li-ion battery. Electrochim. Acta.

[34] Shang Y., Lin X., Lu X., Huang T., Yu A. (2015). Nano-TiO_2_(B) coated LiMn_2_O_4_ as cathode materials for lithium-ion batteries at elevated temperatures. Electrochim. Acta.

[35] Zeng J., Li M., Li X., Chen C., Xiong D., Dong L., Li D., Lushington A., Sun X. (2014). A novel coating onto LiMn_2_O_4_ cathode with increased lithium ion battery performance. Appl. Surf. Sci..

[36] Aziz S., Zhao J., Cain C., Wang Y. (2014). Nanoarchitectured LiMn_2_O_4_/Graphene/ZnO Composites as Electrodes for Lithium-Ion Batteries. J. Mater. Sci. Technol..

[37] Wang H., Han J., Li L., Peng F., Zheng F., Huang D., Lai F., Hu S., Pan Q., Li Q. (2021). Effects of oxidized Ketjen Black as conductive additives on electrochemical performance of the LiMn_2_O_4_@Al_2_O_3_ cathode in lithium-ion batteries. J. Alloys Compd..

[38] Pasqualini M., Calcaterra S., Maroni F., Rezvani S.J., Di Cicco A., Alexander S., Rajantie H., Tossici R., Nobili F. (2017). Electrochemical and spectroscopic characterization of an alumina-coated LiMn_2_O_4_ cathode with enhanced interfacial stability. Electrochim. Acta.

[39] Zhao J., Wang Y. (2013). Atomic layer deposition of epitaxial ZrO_2_ coating on LiMn_2_O_4_ nanoparticles for high-rate lithium ion batteries at elevated temperature. Nano Energy.

[40] Arumugam D., Paruthimal-Kalaignan G. (2011). Electrochemical characterizations of surface modified LiMn_2_O_4_ cathode materials for high temperature lithium battery applications. Thin Solid Films.

[41] Ju B., Wang X., Wu C., Yang X., Shu H., Bai Y., Wen W., Yi X. (2014). Electrochemical performance of the graphene/Y_2_O_3_/LiMn_2_O_4_ hybrid as cathode for lithium-ion battery. J. Alloys Compd..

[42] Jo K.-Y., Han S.-Y., Lee J.M., Kim I.Y., Nahm S., Choi J.-W., Hwang S.-J. (2013). Remarkable enhancement of the electrode performance of nanocrystalline LiMn_2_O_4_ via solvothermally-assisted immobilization on reduced graphene oxide nanosheets. Electrochim. Acta.

[43] Lin B., Yin Q., Hu H., Lu F., Xia H. (2014). LiMn_2_O_4_ nanoparticles anchored on graphene nanosheets as high-performance cathode material for lithium-ion batteries. J. Solid State Chem..

[44] Chen Y., Tian Y., Qiu Y., Liu Z., He H., Li B., Cao H. (2019). Synthesis and superior cathode performance of sandwiched LiMn_2_O_4_@rGO nanocomposites for lithium-ion batteries. Mater. Today Adv..

[45] Bak S.-M., Nam K.-W., Lee C.-W., Kim K.-H., Jung H.-C., Yang X.-Q., Kim K.-B. (2011). Spinel LiMn_2_O_4_/reduced graphene oxide hybrid for high rate lithium ion batteries. J. Mater. Chem..

[46] Li A., Shao Z., Yang S., Li X., Zhang A. (2020). Precipitation synthesis and enhanced electrochemical performance of graphene-modified LiMn_2_O_4_ for lithium-ion batteries. Ionics.

[47] Hummers W.S., Offeman R.E. (1958). Preparation of graphitic oxide. J. Am. Chem. Soc..

[48] Wilamowska M., Kujawa M., Michalska M., Lipińska L., Lisowska-Oleksiak A. (2016). Electroactive polymer/graphene oxide nanostructured composites; evidence for direct chemical interactions between PEDOT and GOx. Synth. Met..

[49] Michalska M., Ziółkowska D.A., Andrzejczuk M., Krawczyńska A., Roguska A., Sikora A. (2017). New synthesis route to decorate Li_4_Ti_5_O_12_ grains with GO flakes. J. Alloys Compd..

[50] Majchrzycki Ł., Michalska M., Walkowiak M., Wiliński Z., Lipińska L. (2013). Graphene oxide-assisted synthesis of LiMn_2_O_4_ nanopowder. Pol. J. Chem. Technol..

[51] Prabaharan S.R.S., Saparil N.B., Michael S.S., Massot M., Julien C. (1998). Soft-chemistry synthesis of electrochemically-active spinel LiMn_2_O_4_ for Li-ion batteries. Solid State Ion..

[52] Ramana C.V., Massot M., Julien C.M. (2015). XPS and Raman spectroscopic characterization of LiMn_2_O_4_ spinels. Surf. Interface Anal..

[53] Nemanich R.J., Solin S.A. (1979). First- and second-order Raman scattering from finite-size crystals of graphite. Phys. Rev. B.

[54] Kawashima Y., Katagiri G. (1995). Fundamentals, overtones, and combinations in the Raman spectrum of graphite. Phys. Rev. B.

